# Cost-benefit analysis of pharmacist interventions over 36 months in a university hospital

**DOI:** 10.11606/s1518-8787.2020054001895

**Published:** 2020-09-24

**Authors:** Maurilio de Souza Cazarim, João Paulo Vilela Rodrigues, Priscila Santos Calcini, Thomas R. Einarson, Leonardo Régis Leira Pereira

**Affiliations:** I Universidade de São Paulo Faculdade de Ciências Farmacêuticas de Ribeirão Preto Ribeirão PretoSP Brasil Universidade de São Paulo. Faculdade de Ciências Farmacêuticas de Ribeirão Preto. Ribeirão Preto, SP, Brasil; II Universidade de Ribeirão Preto Faculdade de Ciências Farmacêuticas Ribeirão PretoSP Brasil Universidade de Ribeirão Preto. Faculdade de Ciências Farmacêuticas. Ribeirão Preto, SP, Brasil; III University of Toronto Leslie Dan Faculty of Pharmacy Toronto Canada University of Toronto. Leslie Dan Faculty of Pharmacy. Toronto, Canada

**Keywords:** Cost-Benefit Analysis, Pharmacy Service, Hospital, Economics, Pharmaceutical, Pharmaceutical Services

## Abstract

**OBJECTIVE:**

To perform a cost-benefits analysis of a clinical pharmacy (CP) service implemented in a Neurology ward of a tertiary teaching hospital.

**METHODS:**

This is a cost-benefit analysis of a single arm, prospective cohort study performed at the adult Neurology Unit over 36 months, which has evaluated the results of a CP service from a hospital and Public Health System (PHS) perspective. The interventions were classified into 14 categories and the costs identified as direct medical costs. The results were analyzed by the total and marginal cost, the benefit-cost ratio (BCR) and the net benefit (NB).

**RESULTS:**

The total 334 patients were followed-up and the highest occurrence in 506 interventions was drug introduction (29.0%). The marginal cost for the hospital and avoided cost for PHS was US$182±32 and US$25,536±4,923 per year; and US$0.55 and US$76.4 per patient/year. The BCR and NB were 0.0, -US$26,105 (95%CI −31,850 − –10,610), -US$27,112 (95%CI −33,160–11,720) for the hospital and; 3.0 (95%CI 1.97–4.94), US$51,048 (95%CI 27,645–75,716) and, 4.6 (95%CI 2.24–10.05), US$91,496 (95%CI 34,700–168,050; p < 0.001) for the PHS, both considering adhered and total interventions, respectively.

**CONCLUSIONS:**

The CP service was not directly cost-benefit at the hospital perspective, but it presented savings for forecast cost related to the occurrence of preventable morbidities, measuring a good cost-benefit for the PHS.

## INTRODUCTION

The most prevalent neurological diseases, especially if considered in more advanced stages, generally require pharmacological treatments, whose use is characterized by complex dosage, potential for interaction with other medication and/or the occurrence of important adverse reactions ^1^^,^^2^ .

Negative events associated with medication treatment are the cause of 5 to 10 % of hospital admissions, and between 50 and 60% of these could be prevented. The responsibilities of the clinical pharmacist in the hospital environment should occur from the moment of patient admission to patient discharge. During the hospitalization period, the daily analysis of clinical evolution and medical prescriptions involve aspects that address the need for indication of pharmacological therapy, the effectiveness, and safety of treatment, among others that may be associated with unfavorable clinical outcomes ^3^^,^^4^ .

At admission and discharge, a medical reconciliation is recommended. This activity refers to the review of current medical prescriptions and evaluation of possible inconsistencies in relation to the patient's medical history, based on prior medical prescriptions. Pharmacotherapeutic interventions can occur at these three moments: hospital admission, during hospitalization, and discharge ^3^^-^^7^ .

Scientific advances and health technologies promote an increase in the population's life expectancy. However, an aging population poses new challenges, as there is a higher prevalence of chronic morbidities that require specialized and complex care ^8^ . This new reality also makes it absolutely necessary to rationalize decision-making processes in order to improve the application of health resources ^9^ . Hospital admissions due to a neurological condition represent, on average, a cost of US$718.00 per patient. If hospitalization has another primary cause, but involves concomitant neurological care, this cost can be up to five times greater ^10^ . Studies suggest the integration of the clinical pharmacist into healthcare teams that provide care to patients with diseases such as epilepsy, Parkinson's disease and multiple sclerosis, improves clinical outcomes and quality of life of patients assisted by means of pharmacist interventions (PI) corresponding to drug related problems (DRP). DRP may culminate in negative results of medication use ^11^^-^^13^ .

Although the clinical benefits resulting from the implementation of clinical pharmacy (CP) at different levels of healthcare are known, there is a gap that refers to the need for data-driven economic analyses of well-designed prospective studies that show the positive economic impact of the clinical pharmacist activities ^14^^,^^15^ . In this way, evidences from benefit-cost studies are important because this design permits to assess the impact of services and programs, measuring different outcomes in monetary indicators, opposite to cost-effectiveness studies that evaluate outcomes depending on the specific clinical indicators or several outcomes by a single indicator, non-monetary, such as years of life saved ^16^ .

In this context, the aim of this study was to evaluate the cost-benefit of the CP service implemented in a Neurology ward from the perspective of the hospital and the Brazilian Public Health System (PHS).

## METHODS

### Study Design

We carried out a cost-benefit analysis of a single arm, prospective cohort study ^12^ . The economic study was carried out from the hospital and PHS perspective, and composed data from patients followed-up over 36-months, from January 2013 to January 2016, without control group. Results were interpreted as marginal cost of pharmacist interventions (the purpose of CP service in Neurology ward) and the CP service cost for implementing at Neurology ward of the hospital. Marginal cost of pharmacist interventions was estimated reasoned with and without CP services regarding pharmacist interventions. Without CP service was the cost condition that considered the hypothesis which the pharmacist interventions would not occur along the patients care at the Neurology ward, and with CP service was the cost condition over the pharmacist interventions occurred, recognized as adhered or not by medical team ^16^^,^^17^ .

### Study Location and Criteria for Patient Inclusion

The base study for this pharmacoeconomic analysis is an open study that started in July 2012, developed at the adult ward of the Neurology Unit of the General Hospital of the Medical School of Ribeirão Preto, Universidade de São Paulo, Brazil (HCFMRP-USP). HCFMRP-USP is a tertiary hospital focused on teaching, researching, and assisting Brazilian Public Health System users ^12^^,^^18^ . The ward has 26 beds with an organizational structure that involves the following subspecialties: neuromuscular diseases, general neurology, epilepsy and movement disorders ^18^ .

In this study, we included a population of neurological patients, individuals of both genders, aged 18 years or more, who were admitted at the adult Neurology Unit of HCFMRP-USP, stayed there for at least 48 hours, and for whom at least one medication was indicated for continued use during hospitalization ^12^ .

### Clinical Pharmacy Service

The patients included were followed-up from the time of admission to discharge. The patients' pharmacotherapeutic follow-up was performed through daily analyses of the medical prescriptions, clinical evolutions, and laboratory tests. Upon detecting a DRP which was adapted from Strand et al. ^26^ , the clinical pharmacist made an intervention alongside the medical team, through manual and electronic medical record. The interventions were classified into 14 categories according to arrangement of the CP service and the DRP detected.

### Sample

Sample size estimate was performed through a prevalence formula and was based on average prevalence of health team adherence to pharmacist interventions, regarding five previous studies carried out in a similar context to our study. We considered a level of significance (α) of 5% for an infinite population; proportion of pharmacist interventions accepted from 73.4% to 98.4%; error of 0.1015. Sample size estimate was performed through a prevalence formula and was based on average prevalence of health team adherence to pharmacists' PI, regarding five previous studies carried out in a similar context to our study. We considered a level of significance (α) of 5% for an infinite population. Therefore, the minimum sample size required would be 134 individuals. All pharmacist interventions were accounted, but when there was no data for measuring the costs, that one was excluded.

### Data Collection

The data were obtained through the inpatient follow-up records used by the pharmacists during follow-up and the hospital electronic system. About each patient, identification and sociodemographic data, as well as information on medication therapy and about morbidities diagnosed were collected. Regarding pharmacological therapy costs related to pharmacist interventions, data were collected on drugs prescribed to patients at the Neurology ward, such as: name of the drug, indication, pharmaceutical form, medicine dosage, route of administration, dose, and period of use. It is noteworthy the costs were estimated as a marginal cost of CP service regarding its interventions.

For each intervention, information was listed as: DRP; intervention performed; moment of intervention (reconciliation at admission, follow-up, or reconciliation at discharge); adherence by the health team to the intervention, data on the medication involved, as described above; pre-intervention cost (without CP service); post-intervention cost (with CP service). In addition, the classification of the clinical conditions or morbidities avoided with each intervention was performed. Classification was obtained reasoned in the International Code of Diseases (ICD 10) and applied to health-related problems avoided by the corresponding interventions performed by the pharmacist ^12^ .

### Costing

Seeking a direct cost analysis we performed the mixed costing technique (micro costing and macro costing). The bottom-up and top-down methods were performed, the first being applied to the direct costs associated with the pharmacist interventions, and the second to the costs associated with co-morbidities/health complications avoided due to the pharmacist interventions, considered as prospective costs ^16^^,^^17^ .

Identified direct medical costs were collected through the computerized system of the hospital (cost data of medical supplies, medication and exams), and assigned to each type of intervention performed. Costs related to the salary of pharmacist, nursing assistant, and nursing professionals were collected at the Human Resources department of the *HCFMRP-USP* . Data on outpatient costs and the respective hospitalizations of the different morbidities conditioned to the clinical conditions covered in the interventions were collected at the Tabwin Datasus^®^ system ^10^ . These morbidity/clinical condition costs composed the estimate of PHS avoided costs by CP service, and it was interpreted like a benefit of pharmacist interventions.

Initially, cost measurement was performed for each intervention. Subsequently, the total sum for each intervention category was performed, then the annual cost and the total cost of the three years of CP service were measured. It should be noted that outpatient and inpatient costs are available for tabulation in disenable files of the PHS Outpatient Information System (SIA-SUS) and the PHS hospital information system (SIH-SUS). These systems are used to obtain data on clinical/administrative performance in Brazilian public health. Data from all Brazilian states, between the months of January to December of 2016 were selected for a statistical analysis and then composed the avoidable clinical conditions/co-morbidities costs in our study.

Furthermore, Tabwin Datasus^®^ data were selected taking into consideration all the chapters of ICD 10, frequency of occurrence, and codes for classified diseases. After, the information was tabulated in the program and these data were exported to the Microsoft Excel 2013^®^ program in which they were systematized in a table containing the values for diseases classified in ICD 10, and their frequency. By estimating the mean of outpatient and hospitalization values for each classification, we evaluated the outpatient/hospitalization cost forecasts related to the clinical conditions/co-morbidities possibly avoided for the PHS, a measurement of predict cost ^10^ .

The cost of the professional to administer the medication was also considered for some interventions, whenever this type of cost had to be applied. Initially, it was measured per minute to compose the cost of the intervention to which it was assigned. The estimate was carried out by means of the average time of preparation of the drug (data collected by a questionnaire applied to the nurses that work at the neurology ward, which used the Delphi method to systematize the information as results) multiplied by the professional cost per minute worked. The 13^th^ salary was added to the annual cost of the professional. The cost of CP service was estimated by expenditures with one clinical pharmacist at the hospital. It was considered the professional cost, since materials and resources for implementing the service are the same existent at the Neurology ward for the common use and they could be shared.

The cost with the professionals was estimated using the data provided by the human resources department regarding each professional's the salary and bonuses. It was considered 30 hours per week for human capital; it was used mean salary pay for a pharmacist; and mean salary pay for a nurse in the state of São Paulo, Brazil. According to the Brazilian Network Health Technology Assessment, the cost sensitivity was 50% more and less than the mean estimated ^19^ .

The cost for the hospital was estimated by the marginal cost of CP service, difference between with and without CP service (without CP service would be a scenario before intervention, which was considered as the prescription or clinical conduct kept itself until the patient discharge; and with CP service was a scenario after intervention considered the changes of the prescription or clinical conduct). Box summarizes the measurement method for the PI costs considered in this study:

**Box:** Estimate method of clinical pharmacy service marginal cost, described for each pharmacist intervention category

Intervention categories	Without clinical pharmacy service	With clinical pharmacy service
Medication introduction	NA	Cost of medication per day x time of use after intervention
Medication Withdrawal	Cost of the medication per day x time of use if there was no intervention	NA
Dose increase	Cost of medication per day x time of use after intervention	Cost of medication per day x time of use after intervention
Dose Reduction	Cost of medication per day x time of use after intervention	Cost of medication per day x time of use after intervention
Replacement	Cost of medication per day x time of use after intervention	Cost of medication per day x time of use after intervention
Administration time adjustment	NA	NA
Administration route change	Average cost of serums and diluents x number of administrations per day x time after + Professional's cost per minute x average time of IV medication administration at the hospital x time after	Cost of medication per day x time of use after intervention
Dosage form change	Cost of medication per day x time of use after intervention	Cost of medication (new dosage form) per day x time of use after intervention
Pharmaceutical form concentration change	Cost of medication per day x time of use after intervention	Cost of medication (new presentation) per day x time of use after intervention.
Infusion rate change	Average cost of serums and diluents x number of administrations per day x time after + professional's cost per minute x average time of IV drug administration at hospital x time after	Average cost of serums and diluents x number of daily administrations x time after + professional's cost per minute x average time of IV medication administration at hospital x time after
Diluent change	Cost of diluent x number of administrations per day x time after	Cost of new diluent x number of administrations per day x time after
Request for examination	NA	Cost of requested examination
Education/information	NA	NA
Other	NA	NA

Clinical pharmacy service marginal cost	Hospital perspective	PHS perspective
	(Cost with clinical pharmacy service by intervention – Cost without clinical pharmacy service by intervention)	(Cost with clinical pharmacy service by intervention – Cost without clinical pharmacy service by intervention) - (outpatient cost of a comorbidity avoided because of the intervention - hospitalization cost of a comorbidity avoided because of the intervention)

NA: not applicable. Note that a negative value for marginal cost means avoided cost for the hospital as well as for the PHS due to clinical pharmacy service, which can be interpreted as benefits. The category “Education/information” was not considered for cost estimate and has not exclusion criteria; the category “Other” was applied to that interventions not listed in the pharmacist form for interventions, then each should be analyzed individually for cost estimate and for procedures. The time after intervention was considered because this way was possible to make more coherent analysis considering that a patient can have the intervention after some days and then there is patient discharge at the day after the intervention. The estimate of professional's cost was made considering the payroll of the hospital to include the initial salary and incentive premium for the work load of 8h / day, one month of vacations, 20 days of work in the month (taken on weekends and holidays), considering the 13th salary, according to labor legislation. The annual average was performed for the nurse, nursing technician and nursing assistant. The annual compensation was divided by worked minutes in the year to get the professional minute cost. The mean time of drug preparation and administration and / or professional procedure was obtained through the Delphi method. The Delphi method is used when the data are not found in the literature with proven evidence or when the data found are controversial, it is carried out from the selection of experts in the subject where through obtaining data from their answers it is sought to predict trends. In this context, the questionnaire is used to identify the respondents' anonymity

After the identification, measurement and valuing of the costs, the time adjustments were made. The year 2015 was used as the basis for the estimate due to data collection. However, the costs were adjusted for the year 2018. For this purpose, the National Consumer Price Index (NCPI) was considered, which is available in the consolidated economic indicators of the Central Bank of Brazil ^19^ . The conversion into US dollars was made using the consolidating exchange rates for 2015 published by the Central Bank of Brazil, 1 dollar = 3.34 Reais ^16^^,^^17^ . The estimate was made as follows in equation 1 :

(1) $$\begin{array}{l} \text{Time Adjustment:}\\ cost\;x(1+[NCPI\;year\;of\;cost])x\ldots (1+[NCPI\;year\;2018]) \end{array}$$

### Analysis

Results were analyzed by total and per patient costs. CP service marginal cost represented the additional cost involved at the pharmacist interventions for production of health facilities ^16^ . The negative marginal cost represents a monetary benefit and positive represents no benefits. The total and annual costs of the interventions alongside with the costs of the CP service comprised the estimate of the indicators of the cost-benefit analysis for the CP service: benefit-cost ratio (BCR) of the CP service and net-benefits of the CP service for the hospital ( equation 2 and 3 )

(2) $$\begin{array}{l} BCR=benefits\;(marginal\;cost\;of\;clinical\;pharmacy\;service)\\ ÷\;cost\;(clinical\;pharmacy\;service)\text{BCR:}\\ BCR=benefits\;(monetary\;from\;intervention\;costs)÷costs\\ \left(Pharmaceutical\;service\right) \end{array}$$

(3) $$\begin{array}{l} \text{Net Benefit (NB):}\\ NB\text{=}benefits\;(monetary\;from\;intervention\;costs)\;-\;costs\\ (Pharmaceutical\;service)\;NB=benefits\;(marginal\;cost\;of\;\\ clinical\;pharmacy\;service)\;-\;cost\;(clinical\;pharmacy\;service) \end{array}$$

For the PHS, the avoided cost of health complications was added to the marginal costs, in the equation, for composing the benefits from the PHS perspective. The indicators of the cost-benefit analysis were interpreted such as: BCR > 1 or NB > 0, the benefits were higher than the costs showing the CP service could save money; and 0 < BCR < 1 or NB < 0, the costs were higher than the benefits regarding the CP service. If the results were 1 and 0 for BCR and NB, respectively, it meant there was no difference between monetary benefits and costs. Additionally, BCR = 0 meant there was no benefit ^20^ .

Sensitivity of BCR and NB results refer to the possible variation of direct costs of CP service, costs with medicines, human resources, which are aggregated to the marginal cost of PC service regarding pharmacist interventions and also, ambulatory and hospitalization costs with co-morbidities to the PHS. Thus, the sensitivity analysis was performed at 10,000 iterations of Monte Carlo simulation for uncertainties of costs and benefits. We used the @RISK software, version 7, of Palisade Corporation^®^ 2015. The results were interpreted for an accuracy of 5% and for adhered and total interventions.

The MINITAB version 17 statistical software was used for descriptive statistics of the estimated costs performed and summarized in the annual mean of three years of this study representation, standard deviation, minimum and maximum values and inter-quartile ranges, and also in histogram and box plot, which was important to define the probability curves of each variable. In this way, Anderson-Darling statistical test was performed, which measures how much a particular distribution fits the data; the lower this statistic, the better the distribution fits the data, the significance level of 1% was considered.

### Ethics

This study is part of the study approved by the Research Ethics Committee of *HCFMRP/USP* (protocol no. 2586/2013), updated in December 2016, CAAE 29175414.8.0000.5440. Available at: http://plataformabrasil.saude.gov.br/visao/centralSuporteNova/consultarProjetoPesquisa/consultarProjetoPesquisa.jsf .

## RESULTS

A total of 334 patients were followed-up by the CP service for 36 months (mean of 112 patients followed-up each year), of which 172 were women. The mean age of the patients was 51±16 years and most patients declared themselves white (n = 279). Of the total of 334 patients followed-up, there was a need for pharmacist intervention for 181 (54 %) patients. Among these, most patients were men (n = 93) and the mean age was 53±16 years.

Most of the pharmacist interventions (86%) occurred during hospitalization, and the other 14% were carried out at admission or discharge. It is noteworthy that of the total interventions, the percentage of acceptance by the health team was 70%. Of the 506 interventions, that with the highest occurrence was the medication introduction, which presented the percentage of 29% of the total number of interventions.

The cost with the professionals was estimated for the mean between Nurse Assistants and Nurses which was US$5.70 per hour at the hospital and US$0.10±0.03 per minute (95%CI 0.08–0.014). Pharmacist cost was US$8,520±850 (95%CI 8,353–12,780), which considered 30 hours of work during the week that was measured for the year.

The total of the interventions resulted in a direct cost of US$3,473 for the hospital over three years, which represented an annual average of US$1,158. The marginal cost was US$182 per year, which represented the marginal cost of US$0.55 per patient/year. The cost avoided for the PHS was US$25,536 per year, US$76.40 per patient/year ( Table 1 ).

**Table 1:** Total and annual costs (US$) broken down by pharmacist intervention adhered to at moments of conciliation and follow-up by the clinical pharmacy service

Intervention category	Cost without CP service	Cost with CP service	CP service marginal cost (SD)	Avoided costs for the PHS (SD)
Medication introduction	0	518	518	26,481
Medication withdrawal	269	0	−269	14,789
Dose increase	243	532	290	10,132
Dose reduction	1,962	1,393	−569	7,695
Medication replacement	27	76	48	4,568
Administration time adjustment	0	0	0	8,449
Administration route change	32	21	−11	12
Dosage form change	353	12	−341	753
Pharmaceutical form concentration change	30	25	−5	178
Infusion rate change	9	10	0.6	513
Examination requirement	0	886	886	3,038
Other	0	0	−0.6	0.6
Total	2,927	3,473	547	76,609
**Cost per year**	**976**	**1,158**	**182±32**	**25,536±4,923**

CP: clinical pharmacy; PHS: Public Health System; Marginal cost: (cost with clinical pharmacy service by intervention – cost without clinical pharmacy service by intervention); SD: standard deviation.

Note: Negative results in the marginal cost mean resource saving for the hospital.

Interventions in the medication conciliation at the hospital did not generate a direct cost impact on the hospital, but they are likely to have an impact on the PHS ( Table 2 ).

**Table 2:** Direct costs (US$) of adhered to and non-adhered to interventions to the hospital and PHS perspective

Intervention	Avoided outpatient cost of co-morbidity	Avoided cost of hospitalization of co-morbidity	Sum of avoided outpatient costs and hospitalization costs	CP service marginal cost (hospital perspective) (SD)	Avoided cost (PHS perspective) (SD)
Adhered	4,073	73,081	77,154	547	76,609
Non-adhered	1,518	32,000	33,518	1,007	32,511
Conciliation adhered to	266	4,352	4,618	0	4,618
Conciliation non-adhered to	128	3,189	3,317	0	3,317
**Total per year**	**1,995**	**36,479**	**39,536**	**518 (83)**	**39,019 (4,755)**
**Per patient year**	**6.0**	**109.2**	**118.4**	**1.5 (0.6)**	**116.8 (21)**

CP: clinical pharmacy; PHS: Public Health System; SD: standard deviation.

Note: The marginal cost from the hospital perspective was estimated as the cost with the intervention minus the cost without intervention. For estimating the avoided cost to the PHS perspective was made: Sum of avoided outpatient and hospitalization costs – marginal costs of CP service). Thus, a positive marginal cost means no benefits of CP service and a positive avoided cost for the PHS means a real monetary benefit. Negative values for the marginal cost indicate that the cost margin was converted in favor of the intervention, characterizing the monetary benefit, or an expense that was avoided.

The BCR and NB obtained from the interventions adhered to and the total of interventions showed, from the hospital perspective, there was no monetary benefit, presenting negative NB, and ratio equal to zero. However, BCR from the perspective of the PHS was 3.0 with NB of US$51,049 and 4.6 with NB equal to US$91,496, considering only the interventions adhered to and the total of interventions, respectively ( Table 3 ).

**Table 3:** Benefit-cost ratio by intervention and summary result based on clinical pharmacy service benefit-cost to the hospital and PHS perspective

Pharmacist interventions	Adhered interventions		Non-adhered interventions		Total interventions	
	Hospital	PHS	Hospital	PHS	Hospital	PHS
Medication Introduction	0.00	3.10	0.00	0.80	0.00	3.90
Medication withdrawal	0.03	1.70	0.03	0.40	0.06	2.20
Dose Increase	0.00	1.20	0.00	1.40	0.00	2.60
Dose reduction	0.07	0.90	0.00	0.20	0.07	1.10
Medication replacement	0.00	0.50	0.00	0.20	0.00	0.70
Administration time change	0.00	1.00	0.00	0.80	0.00	1.80
Administration route change	0.00	0.00	0.00	0.00	0.00	0.00
Dosage form change	0.04	0.09	0.00	0.00	0.04	0.09
Pharmaceutical form concentration change	0.00	0.02	0.00	0.00	0.00	0.02
Infusion rate change	0.00	0.06	0.00	0.00	0.00	0.06
Request for examination	0.00	0.40	0.00	0.10	0.00	0.50
Other	0.00	0.00	0.00	0.00	0.00	0.00

Summarized Analysis	Adhered interventions		Total interventions	
	Hospital	PHS	Hospital	PHS
Clinical pharmacy direct cost (US$)	25,560	25,560	25,560	25,560
Benefit (Cost US$ avoided by the clinical pharmacy service)	0	76,609	0	117,056
Marginal cost (US$)	547	0	1,552	0
**BCR of clinical pharmacy service**	**0**	**3.0**	**0**	**4.6**
**NB (US$) of clinical pharmacy service**	**−26,105**	**51,049**	**−27,112**	**91,496**

BCR: benefit-cost ratio; NB: net benefit; PHS: Public Health System.

Note: Benefit-cost ratio of the interventions considered the marginal cost of the clinical pharmacy service by intervention and clinical pharmacy direct cost (zero means there were no benefits), highlighting that the comorbidity costs avoided were measured not specifically for this public hospital but for the whole PHS, scenery where the hospital is fitted. The benefit-cost ratio equal to zero indicates there was no benefit; Negative net benefit means that costs were over than benefits.

The sensitivity of BCR and NB showed there was no sensitivity for the hospital as the BCR, which had no direct benefit. However, NBs were -US$26,105 (95%CI −31,850–10,610), -US$27,112 (95%CI −33,160–11,720) for adhered and total interventions ( Figure, E and F ). PHS had BCRs of 3.0 (95%CI 1.97–4.94), 4.6 (95%CI 2.24–10.05) and NBs of US$51,048 (95%CI 27,645–75,716), US$91,496 (95%CI 34,700–168,050) for adhered and total interventions, respectively ( Figure, A, B, C and D ).

**Figure 1:**
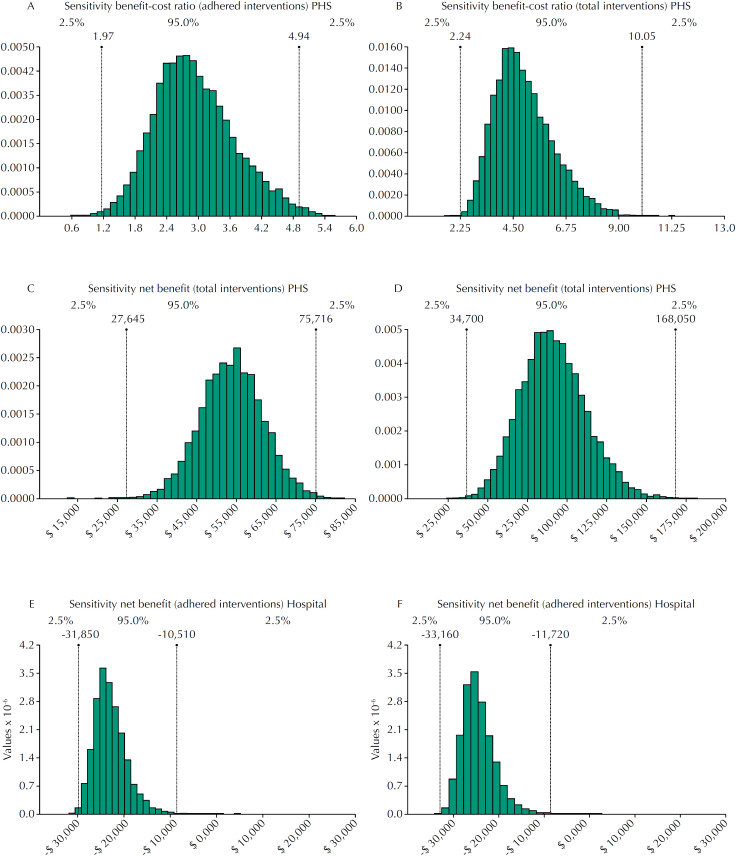
Sensitivity analysis of benefit-cost ratio and net benefit regarding pharmacy's clinical service.

This figure has grade plan because it is requested for this kind of analysis and it is make by the @Risk software. BCR: benefit-cost ratio; NB: net benefit; PHS: Public Health System.

Note: It was performed Monte Carlo simulation for 10,000 iterations and the results were interpreted for 95% of confidence. A) Amplitude of values of Benefit-cost Ratio as adhered interventions for public health system perspective; B) Amplitude of values of Benefit-cost Ratio as total interventions for public health system perspective; C) Amplitude of values of net benefit as adhered interventions for public health system perspective; D) Amplitude of values of net benefit as total interventions for public health system perspective; E) Amplitude of values of net benefit as adhered interventions for hospital perspective; F) Amplitude of values of net benefit as total interventions for hospital perspective.

## DISCUSSION

Regarding the total number of patients included, 54% (n = 181) required one or more pharmacist interventions during the hospitalization period, generating an average of 2.7 interventions per patient, higher than that found by Nunes et al. ^21^ . In that study, 30.4% of the patients attended at the Jamil Haddad National Institute of Traumatology and Orthopedics required the implementation of CP services.

Of the 506 interventions performed by the CP service at the adult ward of the *HCFMRP-USP* Neurology Unit, most interventions were accepted, generating a 70 % adherence rate (n = 354). This suggests pharmacist interventions are well accepted by the health team. A similar rate was identified in the study by Gardner et al. ^22^ , with 71% adherence to pharmacist interventions in psychiatric care and in a multicenter study conducted in France that found an acceptance rate for the intervention performed by pharmacists of 73% ^23^ .

It is important to highlight that in this study we measured the impact of the adhered interventions as the total of pharmacist interventions, without discussing the clinical merit in question, and that this analysis showed us an important difference of approximately US$1,000 in NB for the hospital and, 35 % in BCR and US$40,500 in net benefits for the PHS. In a study carried out in an intensive care unit, 98.4 % of the pharmacist interventions were accepted, which generated savings of US$2,479 in CP service marginal cost ^24^ . The CP service generated a forecast of cost savings for the PHS of US$76,609 and US$117,056, resulting from the achievements of the interventions that were adhered to and total interventions, respectively. Although CP service has not presented a positive economic impact for the hospital, outcomes were not measured. Hence, possible improvements as treatment effectiveness and quality of life could be health benefits to be assessed ^4^^,^^15^ .

The sensitivity of these results showed even with a variation of costs to estimate monetary benefits and CP service costs the maximum NB would still be negative for the hospital, -US$10,610, there would be no monetary benefits at this perspective. However, cost savings could get up to US$48,000 per year at the PHS perspective, which was measured by BCR of 4.6 that could reach 10.5, considering total of interventions. This BCR can be compared to the fourth best strategy around the world, the control of malaria program, which had a BCR of 10.0 in 2012 ^25^ .

The management of pharmacotherapy through pharmacists' interventions, which is difficult and complex when involving inpatients with neurological diseases, specially older ones ^2^^,^^26^^,^^27^ , can seem costly in a simple direct cost analysis. For instance, in this study the prevalence of medicine addition was higher than withdrawal, which certainly reflected on direct hospital costs. In this way, the analysis of the perspectives of the PHS compared to the hospital perspective, showed a significant economic impact that the CP service can cause in resource savings. In CP services developed in specialized environments, it is common for interventions that introduce medication to be the majority since, as well as aiming to reduce morbidity in the short and long term, reflect the managerial action of the pharmacist for patient-centered care ^28^ .

According to the Tabwin Datasus® system, the percentage of hospitalized patients, identified by Chapter VI – Nervous System Diseases of ICD 10 – as secondary diagnosis at the time of admission, was 2.78% in 2016 in Brazil ^10^ . Considering this data, it is possible to extrapolate the results from this study and conjecture that this CP service evaluated would be able to impact on the national reduction of this hospitalization percentage to 2.76%. If this was implemented in the whole hospital and in other hospitals, the national impact would be higher than this difference of 0.02%.

In addition, in the year 2016 there were 15,452 national admissions of adult patients by primary diagnosis of diseases of the nervous system, which cost US$11 million for the PHS ^10^ . Considering this scenario, alongside data from 2014 in the *HCFMRP-USP* neurology ward, which indicated 679 admissions, it is possible to make an analogy extrapolating the results of this study to conjecture that approximately, US$1.8 million per year could be saved for the PHS with the implementation of this CP service at the Neurology wards at national level.

This scenario indicates substantial savings for the PHS, which could result in reallocation of resources. Brazil is the largest country in terms of population dependent on the PHS. It is noteworthy that resources used in public health are lower than the ones used in developed countries and approximately 72% of Brazilians depend on the PHS to access healthcare ^29^ .

According to the national health survey carried out in 2013 by the Brazilian Institute of Geography and Statistics of the total number of residents in Brazil, about 6.0% had a hospitalization of 24 hours or more, and of this percentage, approximately 66.0% were hospitalized at the PHS ^29^ . Thus, the implementation of the CP service can generate several benefits for the health sector, being able to reduce the hospitalization time of patients, which would result in lower expenses with medications, costs related to hospitalization, and could contribute to improve the rational use of health resources and to assist in the promotion of humanized and patient-centered care ^30^ . This is an important front to be followed within the broad perspective of healthcare, which considers the individual in all their psychosocial aspects as the target of care ^31^^,^^32^ .

There were limitations in this study as the absence of clinical records of the hospital for economic evaluation, which was responsible for reducing 12% of the sample, but even in this condition the sample was 2.5x higher than the sample plan number, approximately. Results related to clinical conditions/co-morbidities possibly avoided for the PHS had been measured by the analysis of non-treated conditions (performed by experts in medicine and CP) and had not been obtained by a conventional way like a prospective study. However, we were careful with this influence and we have considered the variation of ambulatory and hospitalization costs according to real data from Datasus. Then we believe it was reasonable for presenting robust results because pharmacist interventions that are reasoned in DRP, as in this study, are actions to improve effectiveness and avoid future health complications or co-morbidities ^8^^,^^26^ to PHS perspective, as well as to public hospitals, although it was not measured due to this study design. Furthermore, this method may be considered efficient when we think about research efficiency in cost terms as the time for developing the study. It is noteworthy, the results showed in this study are capable of foster pharmacoeconomic studies as the management of the health resources and its applicability, for instance, the saving costs for PHS could cover the costs with the CP service and its marginal cost for the hospital.

## CONCLUSION

Results suggest the CP service has been well accepted by the Neurology ward health team. In this way, the CP service can be able to promote improvements in pharmacotherapeutic management at the Neurology health care. Moreover, the economic impact measured was positive for the PHS, which presented a good cost-benefit ratio and net monetary benefit. However, CP service did not represent a good cost benefit for the hospital, since there were higher costs than monetary benefits, viewed by PC service marginal cost. It is relevant to consider the amount saved for PHS could cover the hospital costs with the CP service and its marginal cost.
